# Effect of Eye Movement and Body Position on Optic Nerve Sheath Width

**DOI:** 10.18502/jovr.v21.17638

**Published:** 2026-10-22

**Authors:** Duy Huyhn, Simran Ohri, Viraj Garg, Jason Zehden, Jessica Duddleston, Christopher Wiesen, Maja Kostic, David Fleischman

**Affiliations:** ^1^Brody School of Medicine at East Carolina University, Greenville, North Carolina, USA; ^2^Department of Ophthalmology, University of North Carolina, Chapel Hill, North Carolina, USA; ^3^Odum Institute for Research in Social Science, University of North Carolina at Chapel Hill, Chapel Hill, North Carolina, USA

**Keywords:** Cerebrospinal Fluid, Glaucoma, Optic Nerve Disease, Optic Nerve Sheath, Orbital CSF Dynamics

## Abstract

**Purpose:**

Orbital cerebrospinal fluid (CSF) dynamics are poorly understood but are postulated to play a role in several optic nerve pathologies. This study aims to evaluate the effects of eye movement and body position on perioptic CSF clearance by optic nerve sheath width (ONSW) measurement.

**Methods:**

Healthy adult patients were recruited prospectively from an outpatient ophthalmology clinic at an academic center. B-mode ultrasound was used to measure optic nerve sheath width (ONSW) 3 mm posterior to the optic disc after 5 minutes of no eye movement (–EM) and 1 minute of eye movement (+EM) in three body positions: upright, supine, and prone. CSF clearance is the difference between ONSW +EM and –EM in each body position.

**Results:**

Thirty-eight adults with healthy optic nerves were recruited. ONSW –EM were significantly wider than +EM in all three positions (*P* = 0.002, 0.024, 0.002). Body positioning only significantly affected ONSW –EM (*P* = 0.030), while +EM ONSW was not significantly different in any body position, indicating a baseline effect.

**Conclusion:**

Eye movement and body position significantly impact orbital CSF dynamics and ONSW. Prone positioning increased ONSW, highlighting the role of gravity dependence in perioptic CSF clearance. Identifying factors that impact CSF dynamics is imperative to understanding its part in optic nerve disease.

##  INTRODUCTION

Cerebrospinal fluid (CSF) plays an increasingly recognized role in ocular health and optic nerve disease. Conditions such as glaucoma, spaceflight-associated neuro-ocular syndrome (SANS), including hypothesized alterations in the ocular glymphatic system during microgravity exposure,^[1]^ and postoperative posterior ischemic optic neuropathy may all be influenced by alterations in CSF dynamics. The optic nerve is surrounded by the optic nerve sheath, which contains all three meningeal layers. Its subarachnoid space is contiguous with that of the brain, allowing orbital CSF to interact directly with intracranial CSF pathways.^[2]^

CSF movement is essential for central nervous system (CNS) waste clearance and is mediated by the glymphatic system, a recently described perivascular and intraparenchymal network.^[2]^ Impairment of this clearance mechanism has been implicated in neurodegenerative conditions and may also contribute to optic nerve pathology. In the eye, reduced CSF turnover within the perioptic subarachnoid space (PSAS) could lead to the buildup of metabolic waste, potentially playing a role in the development of glaucomatous damage.^[3,4]^

Studying orbital CSF dynamics is challenging because lumbar CSF pressure does not necessarily reflect orbital pressure, and most human data are indirect. Animal studies have suggested that gravity and eye movement influence CSF flow. In a porcine model, dependent CSF migration was observed during anesthesia-induced ocular immobility, implicating eye movement as a possible driver of fluid clearance.^[5]^

Liu and Kahn used cadaveric and laboratory models to demonstrate that eye movements promote CSF egress from the PSAS in humans.^[6]^ This suggests that natural eye movements—during both wakefulness and rapid eye movement (REM) sleep—may play a critical role in orbital CSF clearance.^[6,7,8]^ Additional studies have shown that body position influences orbital CSF, likely due to hydrostatic gradients.^[6,9,10,11,12]^

Taken together, these studies suggest three key points: CSF flow into the orbit is gravity-dependent; ocular immobility leads to CSF accumulation; and eye movement facilitates CSF clearance. These dynamics may help explain how waste is removed from the optic nerve and replaced with freshly circulated CSF.

Noninvasive measurement of optic nerve sheath width (ONSW) via B-mode ultrasonography provides a surrogate for evaluating changes in orbital CSF volume.^[11,12,13]^ Measuring ONSW in different body positions, with and without eye movement, offers a practical method for assessing these influences *in vivo*.

This study aims to establish baseline data on the effect of eye movement and body position on ONSW in healthy, non-glaucomatous adults. These findings may provide a foundation for understanding how CSF dynamics contribute to optic nerve vulnerability in disease.

##  METHODS

This is an experimental study conducted at a single outpatient ophthalmology clinic. The study was approved by the Institutional Review Board (IRB) and adheres to the tenets of the Declaration of Helsinki. All participants gave informed consent.

### Study Population

Study participants were healthy adult volunteers. Exclusion criteria were current optic nerve edema or papilledema, history of previous surgery that could affect the optic nerve, diseases affecting the optic nerve or disc (such as glaucoma, optic neuritis, ischemic and compressive optic neuropathy), inability to keep eyes in a fixed position for 5 minutes, inability to lie in a supine or prone position, pregnancy, or patients who were missing one or both eyes or optic nerves.

### Measurements

Ultrasound was used for ONSW measurements due to low cost, nonionizing radiation, and feasibility in an outpatient clinic setting.^[12]^ Despite the accuracy of MRI, the high cost and multiple measurements required made it a less reasonable choice for this study. Measurements were obtained with a Sonosite M-TurboⓇ (Bothell, WA, USA) portable ultrasound with a 14-5MHz probe in B-mode.

Optic nerve measurements were taken after no eye movement (–EM) and eye movement (+EM) in the upright, supine, and prone positions. For –EM measurements, subjects were instructed to look straight ahead for 5 minutes. For +EM measurements, patients moved their eyes continuously in all directions for 1 minute.

The optic nerve and sheath were identified in the transverse plane at a depth of 4.3 cm. The width of the optic nerve and sheath was measured 3 mm posterior to the optic disc, as this is the location of maximal pressure change along the optic nerve.^[14]^

All measurements were performed by a single trained examiner to reduce interobserver variability. However, the examiner was not masked to experimental conditions, and no formal inter-rater reliability metrics, such as intraclass correlation coefficients (ICC) or kappa statistics, were collected, which we acknowledge as a study limitation.

### Power and Sample Size

The system tolerance of the M-turbo ultrasound system is within 0.3 mm. The study was powered to detect a 0.3 mm difference or more between measurements based on a standard deviation of 0.6 mm in normal adults. Given a minimum correlation of 0.5 between measurements, this study required a sample size of at least 34 patients for a power of 80% to detect a difference of half a standard deviation at 0.05 significance.

### Statistics

SAS version 9 (Cary, NC, USA) was used to determine sample size calculations and to compare ONSW measurements. Two-tailed paired *t*-tests were performed, and *P*-values were adjusted for multiple comparisons using the adaptive Holm method to control family-wise Type 1 error. SPSS version 27.0 (IBM Corp., Armonk, USA) was used for logistic regression analysis evaluating the effect of age, gender, race, or BMI on individual measurements. While the Holm method appropriately reduced false positives, we acknowledge that this conservative correction may have obscured biologically meaningful trends. Nominal *P*-values approaching significance (e.g., prone vs. supine –EM) were noted for potential further study.

##  RESULTS

### Demographics

Informed consent was obtained for a total of 48 participants who met criteria for the study. After data collection, 10 patients were excluded due to poor imaging that would preclude reliable measurement of study variables. Average patient age was 43.7 ± 14.8 years, and average BMI was 27.5 ± 6.90 kg/m^2^. There were 12 (31.6%) males and 26 (68.4%) females. Twenty-four adults were White (63.2%), eight were Black (21.1%), and six were Asian (15.8%). There were measurements of 21 (55.3%) right eyes and 17 (44.7%) left eyes [Table 1].

There were no significant correlations between age and the average optic nerve width (ONW; R = –0.285, *P* = 0.082) or ONSW (R = –0.063, *P* = 0.708) [Table 2]. There were no significant correlations between BMI and the average ONW (R = 0.004, *P* = 0.983) or ONSW (R = 0.097, *P* = 0.563). In addition, there were no correlations between age or BMI and ONSW or ONW in all positions with and without eye movement. Kruskal-Wallis tests comparing the average ONSW and average ONW for each patient to race were not significant. Mann–Whitney U tests comparing the average ONSW and average ONW for each patient to gender and eye laterality were not significant.

**Table 1:** A summary of the demographics of the study population along with optic nerve and sheath measurements compared between gender, race, and eye laterality.

		**Mean**	**Standard deviation**	**Median**	**Range**
Age		43.7	14.8	39	21-75
BMI		27.50	6.90	25.19	19.12-49.61
	*N* (%)	Sheath average ± Std. Dev.	Nerve average ± Std. Dev	Sheath median (range)	Nerve median (range)
Gender					
Male	12 (31.6%)	5.90 ± 0.70	3.37 ± 0.73	5.8 (3.5-7.5)	3.2 (2.3-5.6)
Female	26 (68.4%)	5.69 ± 0.90	3.19 ± 0.56	5.5 (3.7-8.1)	3.1 (1.7-5.1)
Race					
Caucasian	24 (63.2%)	5.67 ± 0.88	3.17 ± 0.58	5.5 (3.5-8.1)	3.1 (1.7-5.1)
African American	8 (21.1%)	5.85 ± 0.75	3.32 ± 0.59	5.9 (4.1-7.4)	3.3 (2.3-4.8)
Asian	6 (15.8%)	6.09 ± 0.66	3.53 ± 0.74	6.1 (4.5-7.5)	3.4 (2.6-5.6)
Laterality					
Right eye	21 (55.3%)	5.86 ± 0.81	3.29 ± 0.67	5.9 (3.7-7.8)	3.1 (2.2-5.6)
Left eye	17 (44.7%)	5.66 ± 0.87	3.20 ± 0.58	5.5 (3.5-8.1)	3.1 (1.7-5.1)

**Table 2:** Summary of optic nerve and sheath widths in each body position with (+EM) and without (–EM) eye movement.

	**Sheath average ± Std. Dev.**		**Nerve average ± Std. Dev.**	**Sheath median (range)**	**Nerve median (range)**
Overall	5.72 ± 0.68	3.22 ± 0.52		5.6 (3.5-8.1)	3.1 (1.7-5.6)
Upright –EM	5.77 ± 0.77	3.15 ± 0.58		5.7 (4.4-7.2)	3.2 (1.9-4.3)
Upright +EM	5.40 ± 0.93	3.11 ± 0.57		5.4 (3.5-8.1)	3.0 (1.7-4.6)
Supine –EM	5.82 ± 0.85	3.24 ± 0.61		5.7 (4.2-7.7)	3.1 (2.4-4.8)
Supine +EM	5.51 ± 0.86	3.31 ± 0.66		5.3 (3.7-7.5)	3.2 (2.2-5.1)
Prone –EM	6.09 ± 0.77	3.38 ± 0.73		6.1 (4.9-7.8)	3.2 (2.2-5.6)
Prone +EM	5.66 ± 0.85	3.31 ± 0.62		5.5 (4.3-7.7)	3.1 (2.5-5.5)
EM, eye movement.					

**Table 3:** Summary of two-tailed, paired *t*-tests comparing each body position with (+EM) and without (–EM) eye movement to each other with the mean difference (mm) and 95% confidence interval (CI) and *P*-values.

**Pairings**	**Mean Difference (mm) [95% CI]**	***P*** **-value**
Upright –EM to Upright +EM	0.38 (0.17 to 0.59)	0.002
Supine –EM to Supine +EM	0.31 (0.04 to 0.58)	0.024
Prone –EM to Prone +EM	0.42 (0.21 to 0.63)	0.002
Upright –EM to Supine –EM	0.00 (–0.25 to 0.25)	1.000
Upright –EM to Prone –EM	0.26 (0.01 to 0.51)	0.030
Supine –EM to Prone –EM	0.26 (–0.04 to 0.56)	0.088
Upright +EM to Supine +EM	0.09 (–0.17 to 0.35)	0.880
Upright +EM to Prone +EM	0.00 (–0.25 to 0.25)	1.000
Supine +EM to Prone +EM	0.15 (–0.24 to 0.54)	0.452

### Measurements

The overall average ONW was 3.22 ± 0.52 mm and average sheath width was 5.72 ± 0.68 mm [Table 2].

Two-tailed, paired *t*-test comparison corrected for family-wise error rate showed that ONSW –EM measurements were significantly wider than +EM in upright, supine, and prone positions:

• Upright –EM versus +EM: 0.38 mm difference (95% CI: 0.17 to 0.59 mm, P = 0.002);
• Supine –EM versus +EM: 0.31 mm difference (95% CI: 0.04 to 0.58 mm, P = 0.024); and
• Prone –EM versus +EM: 0.42 mm difference (95% CI: 0.21 to 0.63 mm, P = 0.002) [Table 3].

When comparing ONSW –EM between body positions:

• Prone –EM was significantly wider than Supine –EM by 0.26 mm (95% CI: 0.01 to 0.51 mm, P = 0.088) and
• No significant differences were found between Upright –EM and Supine –EM (P = 1.000) or between Upright –EM and Prone –EM (P = 0.088).

There were no significant differences between ONSW when comparing +EM between all three body positions [Table 3]. Trends in ONSW measurements by position and eye movement status are illustrated in Figure 1. Individual participant changes from –EM to +EM are shown in Figure 2.

##  DISCUSSION

There were more than twice as many females as males participating in the study; however, ONSW did not significantly differ between genders. This is consistent with results of multiple other studies analyzing normal ranges for optic nerve and sheath widths, although Ertl et al found males had significantly wider ONS than females.^[15,16]^

**Figure 1:**
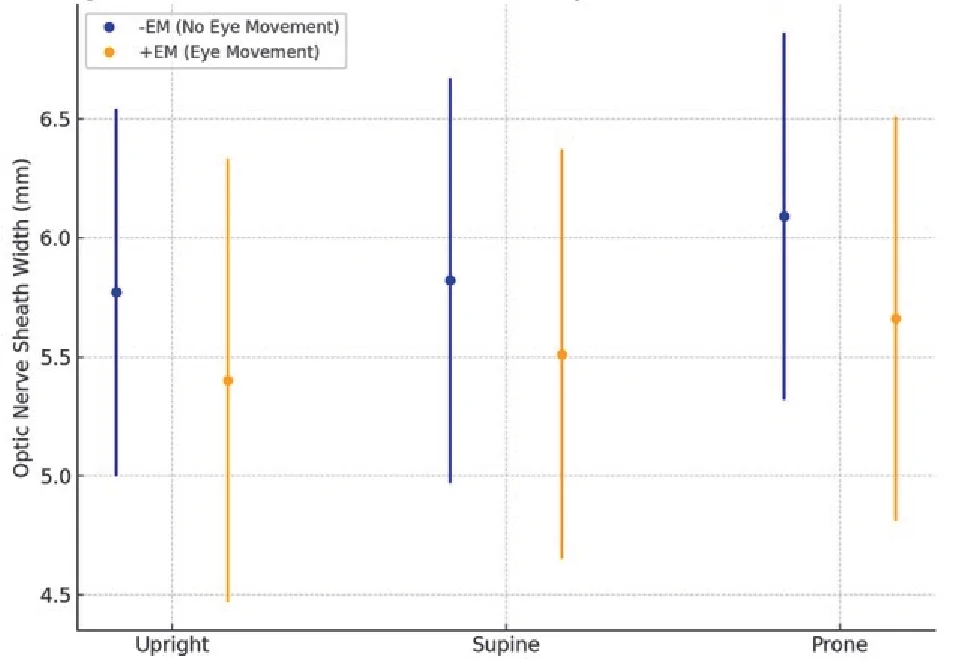
Mean optic nerve sheath width (ONSW) measurements with standard deviations across body positions (Upright, Supine, Prone) and eye movement conditions. Blue markers indicate –EM; orange markers indicate +EM. –EM, no eye movement; +EM, eye movement.

**Figure 2:**
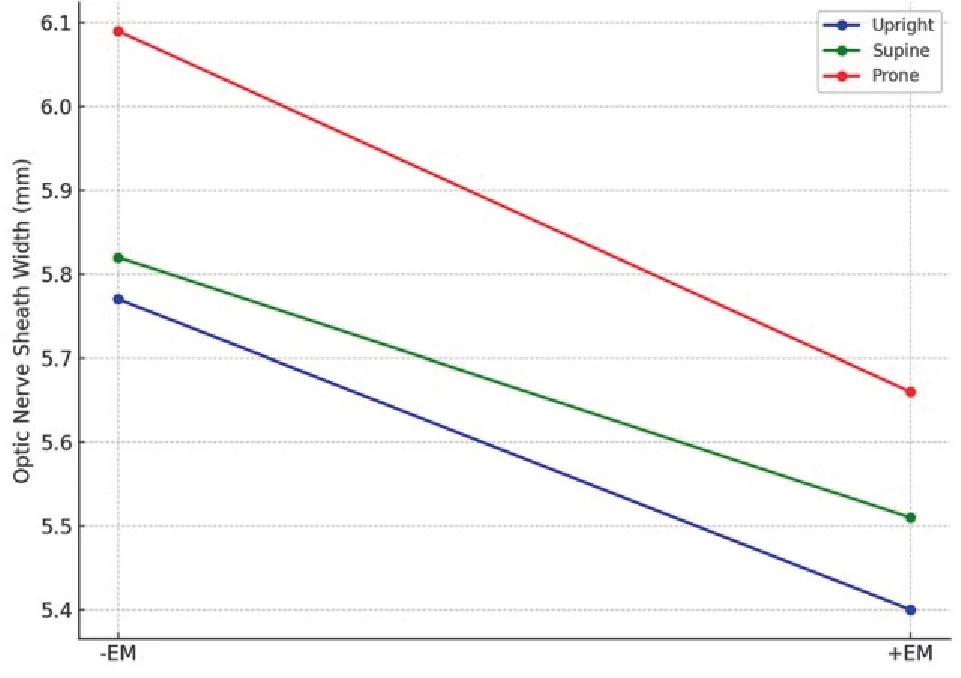
Mean optic nerve sheath width (ONSW) change between no eye movement (–EM) and eye movement (+EM) for each body position (Upright, Supine, Prone). Trend lines demonstrate consistent ONSW reduction after eye movement across positions.

While our findings did not demonstrate statistically significant differences in ONSW between gender or racial groups, the cohort was predominantly female (68.4%) and White (63.2%). Subgroup analyses were underpowered, especially for non-White participants and male subjects, limiting our ability to detect potentially meaningful demographic differences. These nonsignificant findings may represent Type II errors due to small subgroup sizes. As such, these results should be interpreted with caution. Future studies with larger and more diverse populations are needed to evaluate whether CSF dynamics differ meaningfully across sex or racial groups.

Our results show that eye movement significantly reduces ONSW across all positions, supporting the hypothesis that ocular motility promotes CSF clearance in the PSAS. This aligns with cadaveric work by Liu and Kahn^[6]^ and supports the hypothesis that eye movement may facilitate glymphatic-like clearance within the PSAS, particularly during REM sleep. However, while the physiologic plausibility is strong, direct evidence linking eye movement, CSF clearance, and glymphatic activity in the orbit remains lacking. Glymphatic function increases during Stage III sleep, and the subsequent REM phase may help remove CSF-borne waste products.^[17]^

We observed no significant differences in +EM ONSW across body positions, suggesting a stabilizing effect of eye movement on CSF volume. This trend is visually represented in Figure 2, which demonstrates a consistent decrease in ONSW across all body positions following eye movement.

Romagnuolo et al,^[18]^ similarly reported no significant differences in ONSW between supine, Trendelenburg, and reverse Trendelenburg positions, which differs from our observation of increased ONSW during prone immobility. We respectfully note, however, that their protocol did not enforce ocular immobility prior to imaging, whereas our design required 5 minutes of voluntary fixation before –EM measurements. This methodological difference—particularly regarding pre-imaging eye movement—may account for the differences observed. Romagnuolo et al's findings provide important context and highlight the importance of standardized protocols when assessing positional effects on ONSW.

Future studies should explore whether the duration of eye movement or immobility modulates these effects.

This study found that CSF accumulates and results in enlargement of the ONSW with as little as 5 minutes of ocular immobility. Prone surgery is significantly longer than this and may lead to congestion of the PSAS. In conjunction with intraoperative arterial hypotension and iatrogenic anemia, this may contribute to development of ischemic optic neuropathy, a rare complication of non-ocular surgery.^[19]^

Prone positioning during eye immobility resulted in the greatest ONSW, consistent with gravity-dependent CSF pooling. This supports prior porcine and imaging studies, as well as recent ultrasound findings in prone surgical patients, demonstrating hydrostatic influence on orbital CSF dynamics.^[5,20,21]^ The relative differences in ONSW across body positions with and without eye movement are illustrated in Figure 1. Notably, the prone –EM condition showed the highest mean ONSW (6.09 +/– 0.77 mm), supporting the concept of gravitational accumulation of CSF when eye movements are suppressed.

When comparing body positions, prone –EM measurements were wider than supine –EM by 0.26 mm; however, following Holm correction, this difference narrowly missed statistical significance (*P* = 0.08). While this finding suggests a possible gravitational pooling effect, the absolute magnitude of change was small and within the precision limits of ultrasound measurement. Its clinical significance—particularly regarding ischemic optic neuropathy risk—remains speculative and warrants further investigation with larger sample sizes and longer-duration studies.

This study's mean ONSW measurements (5.72 ± 0.68) were wider than in other studies evaluating ONSW. Vaiman et al found ONSW in healthy patients ranged from 3.55 ± 0.82 mm to 5.17 ± 1.34 mm at various locations within the intraorbital space.^[22]^ Wang et al reported an average ONSW of 4.50 ± 0.54 mm in patients with elevated ICP and 3.50 ± 0.21 mm in patients with normal ICP.^[23]^ This variation among studies may be attributed to different protocols in obtaining measurements, including imaging modality and distance posterior to the optic disc. In addition, our measurements were obtained solely using B-mode ultrasound without direct validation against magnetic resonance imaging or other gold-standard techniques. This methodological limitation may contribute to protocol-dependent variability and should be considered when interpreting our results. Lastly, several of the observed differences in ONSW, such as the 0.26 mm increase between prone and supine –EM conditions, approach the ± 0.3 mm precision limit of the ultrasound system. As a result, these findings should be interpreted cautiously, acknowledging the potential contribution of measurement variability.

This study has several limitations. Eye movement was not standardized, and patients were free to move their eyes in whichever direction and order they chose. Future studies would benefit from standardizing eye movement by having participants follow an object on a screen to create a more uniform condition across patients and minimize variability. Employing pupil tracking during the 5 minutes of –EM would also be beneficial to ensure eye movements were below a certain threshold. In addition, there are trabeculae within the PSAS that may contribute to fluid dynamics, which the scope of this study does not account for.

Although we used a standardized imaging protocol, measurements were performed by a single, unmasked examiner, and interobserver agreement metrics (e.g., ICC or kappa statistics) were not recorded, limiting assessment of reproducibility.

Measurement accuracy is also limited by the technology and tolerance of the ultrasound machine, particularly as this study involves measuring anatomy on a very small scale. All measurements were obtained at a single location, 3 mm posterior to the optic disc. While this site corresponds to the area of maximal pressure transmission along the optic nerve, it does not capture regional variations in optic nerve sheath compliance or CSF dynamics. This constitutes a limitation of our study. Future investigations should utilize multipoint ultrasound assessments or MRI-based validation techniques, including emerging studies utilizing ultra-high field MRI and tear biomarkers to evaluate ocular glymphatic clearance.^[24]^

Moreover, individual physiological variables such as hydration status, caffeine intake, circadian phase, systemic blood pressure, and time of day were not standardized or recorded. These factors are known to influence CSF dynamics and may have introduced additional variability into our measurements. Future investigations should consider controlling for these variables to better isolate the effects of eye movement and body position.

Finally, this study did not include a control group of patients with optic nerve pathologies, which limits direct extrapolation of our findings to diseased populations. We are continuing to study this issue, particularly by investigating highly asymmetric cases of glaucoma, to better understand how perioptic CSF dynamics may differ in pathologic states.

Although the study used fixed times of 1 minute (+EM) and 5 minutes (–EM) to evaluate short-term physiologic changes in orbital CSF dynamics, these durations do not replicate real-world conditions such as prolonged prone-position surgeries or extended sleep cycles. The short intervals were deliberately selected to isolate immediate physiologic effects and minimize confounding variables. Nevertheless, extrapolation to longer-duration clinical scenarios should be approached with caution, and future studies incorporating longer observation periods are warranted. The observation that significant ONSW changes occurred after only minutes of immobility underscores the sensitivity of perioptic CSF dynamics to even short periods of ocular inactivity. Future studies extending immobility periods to better model surgical conditions will be critical for determining whether small ONSW differences translate to clinically relevant outcomes.

In summary, this study demonstrates that both body positioning and eye movement significantly impact orbital CSF dynamics and ONSW. Induction of eye movements facilitates increased CSF outflow from the PSAS, reducing ONSW, while prone positioning increases ONSW and highlights the role of gravity dependence. Given the role they play in the development of optic nerve disease, understanding the factors that impact CSF dynamics is crucial to improving our knowledge of pathology affecting the optic nerve and disc. Although our findings do not establish causality or direct clinical implications, they provide physiologic evidence that supports further investigation into the role of perioptic CSF clearance and glymphatic pathways in optic nerve health and disease.

##  Financial Support and Sponsorship

None.

##  Conflict of Interest

None.
